# A Syngeneic Mouse Model of Epithelial Ovarian Cancer Port Site Metastases

**DOI:** 10.1016/j.tranon.2018.08.020

**Published:** 2018-09-27

**Authors:** Ivy Wilkinson-Ryan, Melissa M. Pham, Petra Sergent, Laura J. Tafe, Brent L. Berwin

**Affiliations:** * Dartmouth-Hitchcock Medical Center, One Medical Center Drive, Lebanon, NH 03756, USA; † Geisel School of Medicine at Dartmouth, One Medical Center Drive, Lebanon, NH 03756, USA

## Abstract

Epithelial ovarian cancer (EOC) is a deadly gynecologic malignancy, but animal models for the study of EOC pathophysiology and drug efficacy are limited. Based on the finding that women with EOC are at risk for metastasis at a trocar site after laparoscopy, we developed a syngeneic murine model of port-site metastasis of EOC. We leveraged the ID8 murine EOC cell line to induce intra-peritoneal tumors in mice. Once durable intraperitoneal tumor was confirmed with bioluminescence imaging, intra-abdominal wall tumors were induced by abdominal wall puncture with a hollow bore needle. This resulted in a robust system in which C57BL/6 mice developed metastatic deposits at a rate of 66.7% ± 10.77; no intra-abdominal wall metastases were seen in control samples (*P* = .0003, CI 41.16–90.84). Immunodeficient NOD SCID gamma mice developed puncture site metastases in 70% ± 10.0 of mice and also had no metastases documented in control sites (*P* = .002, CI 42.24–97.76). In addition we were able to demonstrate the presence of immune infiltrates within the metastatic deposits of C57BL/6 mice via IHC. Therefore, in this study we demonstrate the predictable development of invasive abdominal wall metastases in a syngeneic mouse model of EOC. This model enables studies of the metastatic process and provides a novel system in which to test the effect of therapies on a clinically-relevant model in an immune competent mouse.

## Introduction

Ovarian cancer is the deadliest gynecologic malignancy in North America killing an estimated 14,080 women in 2017 [1]. Epithelial ovarian cancers (EOC) are the most common type of ovarian carcinoma and include serous, endometrioid, mucinous, clear cell, and transitional cell histologies. Among epithelial ovarian cancer, high grade serous carcinoma is the most common histologic subtype [1]. At the time of diagnosis, the majority of women have metastases to peritoneal surfaces such as the omentum, bowel, and diaphragm. Although most women with metastatic cancer respond to initial therapy, approximately 70% of patients with metastatic disease experience a recurrence of their cancer with resultant mortality [1]. In order to study the pathophysiology and perform pre-clinical studies of potential therapies researchers have employed animal models of epithelial ovarian cancer. Current murine models of EOC involve transplatation of human or murine ovarian cancer cells into the peritoneal cavity, ovarian capsule, or subcutaneous spaces or genetic alterations that lead to the development of ovarian cancer in transgenic mice [2], [3], [4], [5], [6], [7], [8], [9]. Researchers' ability to perform preclinical studies of ovarian cancer therapeutics and to study the pathophysiology of the disease is limited to the types of available animal models and thus we developed a novel and clinically-relevant metastatic model to add to the repertoire of available murine ovarian cancer research tools.

In humans, intraperitoneal metastasis of ovarian cancer occurs after the primary tumor is established and tumor cells exfoliate off of the primary tumor into the peritoneal cavity [2]. Women with ovarian carcinoma who undergo laparoscopic surgeries are at risk of developing metastatic tumor deposits within the tract of the laparoscopic trocar, also referred to as port site metastases [10], [11], [12], [13], [14]. This has also been observed in patients with other peritoneal cancers who undergo laparoscopic surgery and is thought to be the result of cells migrating from the peritoneal cavity into the trocar tract during or following surgery [15], [16]. Thus, port site metastases are most frequently observed in humans with intraperitoneal tumor deposits and ascites [12], [14]. Although the pathophysiology of this phenomenon has been investigated via animal models, the reproducibility of this process has not been established in murine models nor has this system been used as an outcome for the study of metastatic EOC [17].

Murine models of EOC typically rely on injection or surgical implantation of human or murine ovarian cancer cells into the peritoneal cavity, ovarian capsule, or subcutaneous spaces to establish tumor [2], [3], [4], [5], [6], [7], [13]. Grafting tumor into the ovarian capsule or subcutaneous tissue frequently fails to result in metastatic disease or ascites due to anatomic barriers to tumor spread [2], [3], [4], [5], [6], [7], [13]. An additional limitation of some murine models is the inability to establish clinically relevant tumors in an immune competent mouse for immunologic studies. Studies of xenographs of human ovarian cancer cells in immunosuppressed mice limit our ability to research the immune features of ovarian cancer and the effect of immune therapies [3], [5]. A patient's immune response to their ovarian cancer and the immune cells within the tumor are known to play an important role in metastasis formation [18]. Syngeneic murine models enable focused studies of the immune system's role in metastasis formation and the effectiveness of immunotherapies at preventing metastasis. The development and application of a model of port site metastasis as a system for studying metastatic EOC allows for the use of a syngeneic mouse to model a clinically relevant and traceable metastatic process. Commonly used syngeneic models include the ID8 murine tumor cell line, which we apply in this study, and *MISIIR* transgenic mice and their derivatives [8], [9], [15]. There is a need for the development of a reliable and reproducible model of metastatic tumor deposit utilizing a syngeneic system.

In this study we describe a novel murine model of the pathophysiologic process that leads to port site metastasis in women with ovarian cancer. We were able to predictably induce a metastatic deposit within the abdominal wall in immune competent and immunocompromised mice using the syngeneic murine ID8 EOC cell line [15]. This metastatic model allows for study of a clinically-relevant metastatic implantation in an immunocompetent mouse and can be used as a secondary outcome for pre-clinical drug studies in mice.

## Methods

### Mice and Cells

C57BL/6 mice were purchased from Charles River (Wilmington, MA). NOD SCID gamma (NSG) mice were purchased from the Dartmouth Mouse Modeling Shared Resource (Lebanon, NH). All animal experiments were approved by the Institutional Animal Care and Use Committee. ID8 murine ovarian cancer cells transduced with pFB-neo-Luciferase (ID8-luc cells) were previously described and selected with 0.8 mg/ml G418 [15], [16].

### Establishment of the Port-Site Model

5×10^6^ ID8-luc cells were injected into the peritoneal cavity via a left lower abdominal wall injection. Mice were imaged for in vivo luciferase activity 3–4 weeks following injection, and thereafter as indicated. Mice with radiographic evidence of intraperitoneal tumor were treated with puncture of the right inferior abdominal wall just medial to the nipple with an 18 gauge hollow bore needle. Control sites were identified in the midline of the upper abdomen remote from the ID8 injection site or the puncture site. Mice were sacrificed 3–4 weeks following abdominal wall puncture using CO_2_ gas per institutional protocols.

### Mouse Imaging

Imaging was performed as a modification of a previously described protocol [19], [20]. Briefly, mice were injected with 200 μL of a suspension of 15 mg/mL D-Luciferin Potassium Salt (Gold Biotechnology, St. Louis, MI) in 9% sodium chloride (Baxter, Deerfield, IL) into the peritoneum via the left lower quadrant. Mice were then anesthetized with isoflurane gas. Images were obtained 10 min after Luciferin injection with the Xenogen VivoVision IVIS Bioluminescent and Fluorescent Imager (PerkinElmer, Waltham, MA).

### Tissue Processing and Pathology

Biopsies of the abdominal wall were obtained immediately upon mouse sacrifice. Abdominal wall hair was removed with Nair™. If a palpable nodule or scar was identified in the right lower quadrant in the expected area of the needle puncture (just medial to the nipple), this was marked with a skin pen. If there was no scar or nodule, the area just medial to the nipple was marked. The anterior abdominal wall including the marked site was then excised using a 5 mm Keyes punch biopsy. Abdominal wall biopsies were taken in the same manner remote for the ID8 injection and contralateral to the puncture site and used as paired control sites. Specimens were placed in 4% paraformaldehyde within marked cassettes. Blocks were processed by the Dartmouth Pathology Core Resource. Specimens were embedded into a paraffin block and oriented such that a skin edge is visible on the slide. Slides were cut at 4 microns, air dried, and loaded onto Akura Tissue-Tek Prisma Autostrainer (Leica Biosystems, Buffalo Grove, IL). Slides were dried for 25 minutes, deparaffinized in Xylene, and hydrated through graduated alcohols to water. Cells were stained with Hematoxylin 2 for five minutes and washed in water. Cells were then washed in bluing agent for one minute then washed in water and then 95% alcohol for 30 seconds. Cells were then stained with Eosin-Y for 30 seconds. Slides were dehydrated in 100% alcohol and cleared with xylene. Slides were then mounted with Tissue Tek mounting medium. Staining and dehydrating materials were obtained from Richard Allen Scientific (Grand Island, NY). Pathology slides were interpreted by LJT, our collaborator within the pathology department, who was blind to specimen origin or treatment group.

### Immunohistochemistry (IHC)

Slides were cut at 4 microns and air dried at room temperature. Staining was performed using the Leica Bond Rx Autostainer and Leica Biosystems reagents (Buffalo Grove, IL). The automated protocol includes baking slides for 30 minutes, dewaxing, antigen retrieval using Epitope Retrieval 2, pH 9.0 solution for 20 minutes at 100 °C. Primary anti- CD3 (Abcam, Cambridge, MA) and CD11b (Abcam, Cambridge, MA) antibodies were incubated for 15 minutes followed by washing. Primary antibody binding is visualized using Leica Bond Refine Detection kit with Dab chromogen and hematoxylin counterstain.

### Statistical Analyses

Plots with means and standard deviations obtained from multiple independent experiments with biological replicates are shown. Sample sizes and statistical significance for each experiment are noted in the text. Statistical analyses were performed using GraphPad Prism 7. Student's T-test was used to compare means between groups, with *P* < .05 considered significant.

## Results

### Induction of Abdominal Wall Metastases

**Figure 1:**
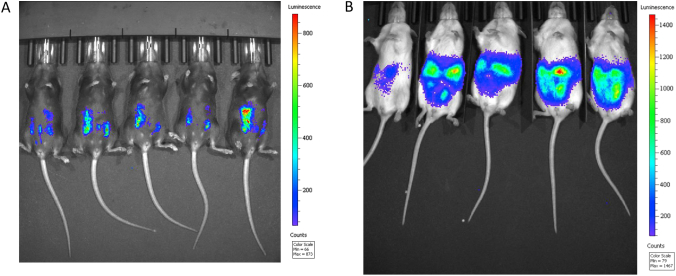
Intraperitoneal ID8 tumor is established at a high frequency in C57BL/6 mice and NSG mice. C57BL/6 mice (A) and NSG mice (B) were injected intraperitoneally with 5×10^6^ ID8-luc cells via the left lower quadrant. Three to four weeks after ID8 injection cohorts of mice were assessed for tumor-associated luminescence using the Xenogen VivoVision IVIS Bioluminescent and Fluorescent Imager (A,B). 83% of C57BL/6 mice and 100% of NSG mice had radiographic evidence of tumor within 4 weeks following ID8 injection.

**Figure 2:**
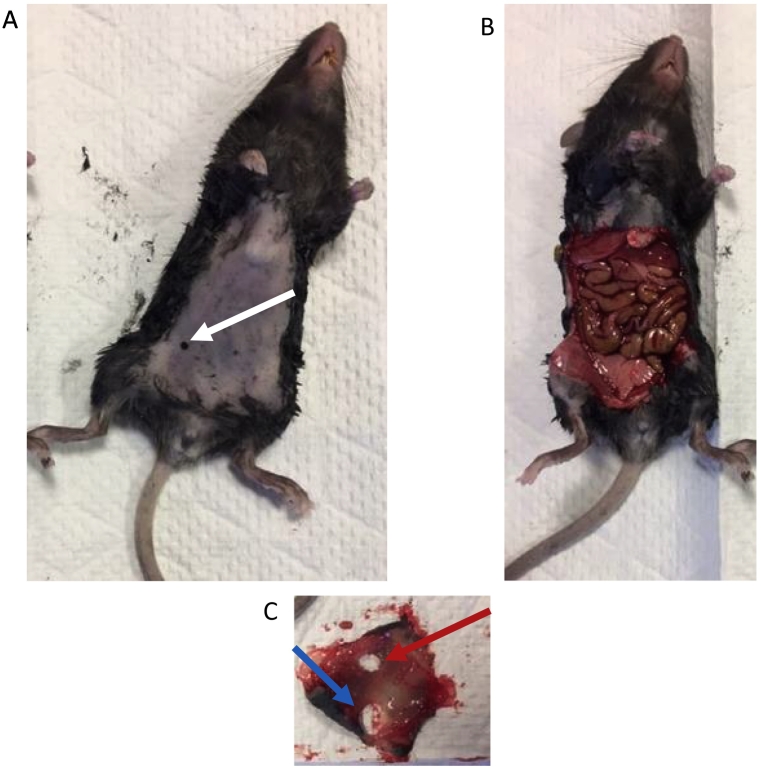
Puncture sites and control sites can be easily processed for histologic evaluation. Once mice were sacrificed and the abdominal hair was removed the puncture site was identified by either a scar or palpable tumor nodule at the injection site (just medial to the nipple). If no scar or tumor nodule was identified, the area medial to the nipple was marked (white arrow, A). The abdominal wall was then excised (B) and a Keyes punch biopsy was used to take a biopsy of a control site remote from the right or left lower quadrant (red arrow) and a biopsy of the puncture site (blue arrow). The black marker was visible transperitoneal if the abdominal wall specimen was placed skin side down on a white surface and this was used to guide the biopsy of the puncture site.

Based on the mechanism of port site metastasis in women with EOC, we generated a metastatic model in both immune-competent and immune-compromised mice. The use of this system in immune competent mice allows for the study of immunologic therapies on metastases formation, whereas use of this system in immune-modified mice allows for the study of the role of specific cell types on the process of metastasis implantation and growth. We employed ID8-luc murine ovarian tumor cells that express luciferase and that are derived from and syngeneic with C57BL/6 mice. ID8-luc tumor cells were transplanted into C57BL/6 and NSG mice, with peritoneal tumor implantation subsequently confirmed by bioluminescence imaging [Figure 1]. Luciferase-positive peritoneal tumors were established in 83% of C57BL/6 and 100% of NSG mice 3–4 weeks following injection of tumor cells. In order to create a port-site lesion, the lower right abdominal walls of mice with intraperitoneal tumor were punctured with a hollow bore 18-guage needle. In order to evaluate for metastasis to the site of puncture, the putative metastatic site (site of puncture) and a control site were biopsied. To do so, the scar or palpable nodule at the puncture site was marked on the intact euthanized mouse. A biopsy from the marked site and a second control site on the abdominal wall were obtained [Figure 2]. Based on this high-yield ID8 system we were able to easily and reliably replicate the process of generating the port site model and collecting puncture site and control site specimens to study the frequency and phenotypes of port site metastases in mice.

### Metastatic Tumors Develop in the Abdominal Wall of Immunocompetent C57BL/6 Mice with Intraperitoneal Ovarian Cancer Following Puncture of the Abdominal Wall

**Figure 3:**
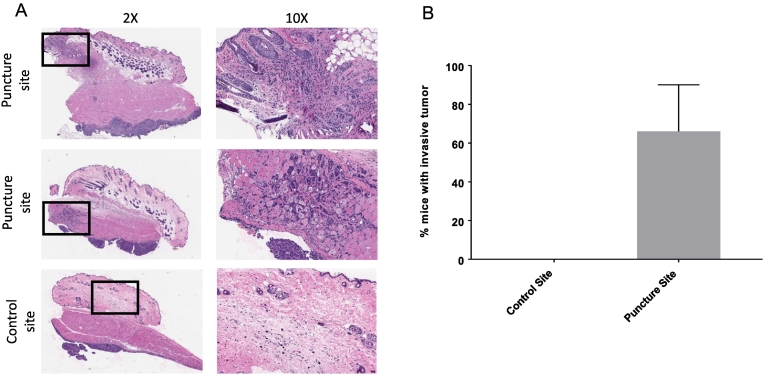
EOC metastases within the abdominal wall at the puncture sites and not the control sites of C57BL/6 mice. (A) Microscopic images of abdominal wall samples from C57BL/6 mice with H + E staining. The first two rows show intra-abdominal wall metastases at the puncture sites. The third row shows an example of a control site with tumor on the peritoneal wall, but no invasive component. Black boxes indicate the area that is enhanced under 10× power and shown on the right. (B) Tumor was identified in 16/24 (66.7% ± 10.77) of puncture site biopsies and 0/14 (0%) of control site biopsies (*P* < .0003, CI 41.16–90.84).

In order to determine the frequency of metastatic intra-abdominal wall tumor and the specificity of metastasis to the abdominal wall puncture site, the puncture site and control site specimens were assessed by pathology for invasive pre-peritoneal tumors within the abdominal wall layers [Figure 3*A*]. In order to quantitate the frequency of port site metastasis in this murine model, puncture site metastases were induced in C57BL/6 mice and biopsies were obtained as described above. Invasive puncture site tumor metastases were identified in 16/24 (66.7% ± 10.77) of puncture site biopsies and 0/14 (0%) of control site biopsies (*P* < .0003, CI 41.16–90.84) [Figure 3*B*]. Therefore, we concluded that the development of intra-abdominal wall metastasis is dependent on abdominal puncture and thus represents a true metastasis from a primary tumor. In addition, we determined that puncture site metastasis could be reproduced at a frequency (66.7%) that is likely to have utility in murine studies on the development of metastases and the effect of therapies on such metastases. Based on these studies, we concluded that intra-abdominal wall metastasis can be induced in a specific area via abdominal wall puncture and subsequently detected by histologic pathology review.

### Metastatic Tumors Develop in the Abdominal Wall of NSG Mice With Intraperitoneal Ovarian Cancer Following Puncture of the Abdominal Wall

**Figure 4:**
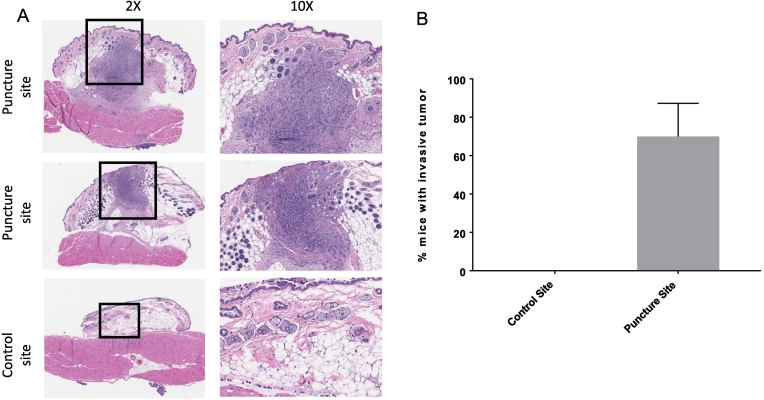
Qualitative and quantitative evaluation demonstrate metastases within the abdominal wall at the puncture sites and not the control sites of NSG mice. (A) Microscopic images of abdominal wall samples from NSG mice with H + E staining. The first two rows show examples of intra-abdominal wall metastasis at the puncture site. The third row shows an example of a control site with peritoneal tumor, but no invasive component. Black boxes indicate the area that is enhanced under 10× power and shown on the right. (B) Tumor was identified in 12/18mice (70% ± 10) of puncture site biopsies and 0/18 (0%) of control site biopsies (*P* < .002, CI 42.24–97.76).

Based on our findings in wild type C57BL/6 mice, we sought to assess if metastasis could be established in immune compromised mice as well. This has relevance because immune modulated mice can be used to elucidate the role of certain immune cell groups on the establishment and growth of metastasis. NSG mice were inoculated with ID8-Luc cells as previously described and sacrificed 3–4 weeks following abdominal wall puncture. The biopsy specimens were processed and H + E slides were reviewed with pathology [Figure 4*A*]. Similar to the immunocompetent C57BL/6 mice, NSG mice had intra-abdominal wall tumor metastases specific to the puncture site. In total, tumor was identified in 12/18 (70% ± 10.0) NSG puncture site biopsies and 0/18 (0%) of control site biopsies (*P* < .002, CI 42.24–97.76) [Figure 4*B*]. Thus, we determined that puncture site metastasis can be established and replicated in immune compromised mice. This now enables the study of the role of various immune components in establishment of port site tumor metastasis by comparing frequencies and rates of metastasis in mice with various immune deficiencies and the contributions of specific subsets of immune cells.

### Metastatic Intra-Abdominal Wall Tumors are Infiltrated With Immune Cells

**Figure 5:**
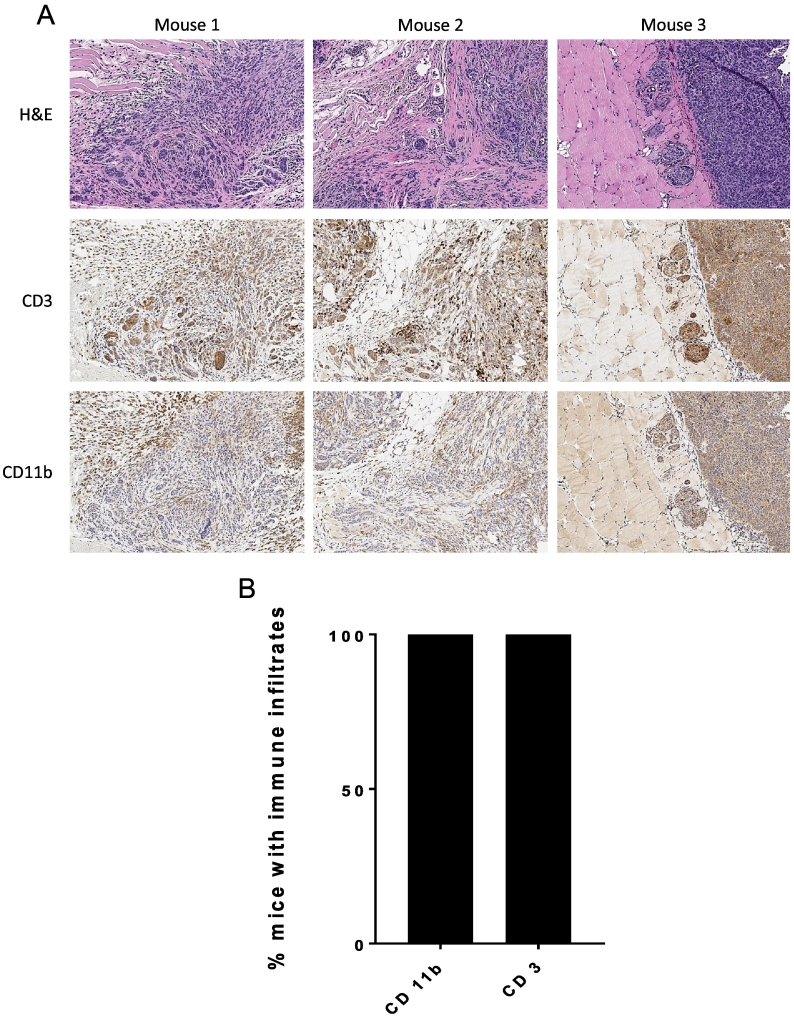
T-cells and leukocytes infiltrate the stroma of puncture site metastases. (A) Immunochemistry of puncture site metastases in three different C57BL/6 mice showing H + E staining (top), anti-CD3 staining of T cells (middle), and anti-CD11b staining of leukocytes (bottom), all taken at 10× power. (B) In all four samples stained (100%) from different C57BL/6 mice there was evidence of intratumoral and/or tumor stroma infiltration of CD3^+^ cells and CD11b^+^ cells.

In order to expand our understanding of how puncture site metastasis can be used for immunologic studies, including potential immunotherapies, we performed immune staining to detect the presence of immune cell populations within the metastasis. Abdominal wall specimens with evidence of invasive disease as assessed by H + E were stained with anti-murine CD3 and CD11b antibodies in order to detect T cells and leukocytes, predominantly macrophages, respectively. Robust populations of CD3+ cells and CD11b + cells were detected within the stroma surrounding metastatic intra-abdominal wall tumor [Figure 5*A*]. Evidence of CD3^+^ and CD11b^+^ cells were identified in 4/4 samples tested (100%) [Figure 5*B*]. Based on these findings we determined that T-cells and leukocytes infiltrate the abdominal wall puncture site metastases. The presence of immune cells within the stroma of puncture site metastases confirms the potential utility of this model for studying the immune related mechanism of tumor cell infiltration and implantation that promotes and facilitates metastases.

## Discussion

Advancements in the study of EOC require development of clinically relevant animal models. Here we describe the establishment and reproducibility of a port site metastasis model of EOC based on the clinical pathophysiology of port site metastasis. The advantage of the model described in this study is that it can be used in an immunocompetent mouse and therefore can be used to study the role of immune infiltrates in the development of port site metastasis as well as the efficacy of new immunotherapies. To develop this model we leveraged ID8 cells which are derived from C57BL/6 mice and are therefore syngeneic to mice on a BL/6 background. Moreover, ID8 tumors have been widely used in syngeneic murine ovarian cancer models [3]. Based on our findings, syngeneic puncture site metastasis can be used to study the role of immune cells in metastasis formation and the effectiveness of immunotherapies [18].

Of note, the abdominal wall puncture site metastasis model documents reproducible establishment of a primary intraperitoneal tumor followed by secondary development of a metastatic site at a predictable location. Thus, this system closely mirrors the process of port site tumor metastasis in humans and allows for tracking of a developing metastasis by inducing the tumor in an identifiable and reproducible location. We utilize a transplantable murine EOC cell line. Another syngeneic model to which researchers could potentially apply the port site model is the MISIIR transgenic mice which develop spontaneous syngeneic murine ovarian malignancy and frequently form intraperitoneal metastasis. [8], [9] Overall, our model closely reflects the mechanical process of metastasis described in women with EOC who undergo laparoscopic surgery [2], [3], [4], [5], [7], [8], [9], [13].

In summary, the puncture site mouse model of port site metastasis represents a novel tool that can be used in animal studies of ovarian cancer. Importantly, this model mirrors a clinical process well described in ovarian cancer patients and is easily reproducible in both wild type (immunocompetent) and genetically modified (immunodeficient) mice. Specifically, we propose that this model can be used as a secondary outcome for therapeutic studies in mice or for mechanistic studies of tumor metastasis. Currently, EOC patients are in need of targeted, efficacious therapies including immune therapies. Models such as this one will be valuable to assess both the biological underpinnings and the potential efficacy of targeted therapies in preclinical studies.
